# Emerging Pharmaceutical Treatments of Novel COVID-19: A Review

**DOI:** 10.7759/cureus.8260

**Published:** 2020-05-24

**Authors:** Azhar Hussain, Jasndeep Kaler, Arun Kumar Dubey

**Affiliations:** 1 Healthcare Administration, Franklin University, Columbus, USA; 2 Medicine, Xavier University School of Medicine, Oranjestad, ABW; 3 Pharmacology, Xavier University School of Medicine, Oranjestad, ABW

**Keywords:** covid-19, sars-cov-2, pharmacology, infectious disease, antivirals, antimicrobials

## Abstract

As a new decade began, COVID-19 quickly gained importance as it became the cause of the current global pandemic. Research has been focusing on studying the structure of SARS-CoV-2 and investigates possible pharmaceutical approaches. With the number of cases increasing every day, globally, multiple drugs are being researched as possible candidates. Although multiple drugs show promise in the treatment of COVID-19 via either inhibiting viral replication or preventing fusion of the virus to the ACE2 receptors, further investigation is still warranted and necessary before the admission of any type of pharmaceutical agent. Furthermore, several supplements have also been documented in being utilized as treatment of COVID-19. The exact mechanism and efficacy of current candidate drugs are still being explored through clinical trials. Despite the advancements in current research with emerging treatments, social distancing and engaging in preventative measures remains crucial to attempt to prevent the occurrence of more cases and deaths, worldwide. This review explores various drugs and their mechanism of action which are either currently being used in clinical trials or may be used in the future for the treatment of COVID-19.

## Introduction and background

Since the emergence of the virus in China in December of 2019, severe acute respiratory syndrome coronavirus 2 (SARS-CoV-2) has spread across the globe resulting in the current global pandemic. As of March 12, 2020, COVID-19 has been confirmed in 125,048 people worldwide, carrying a mortality rate of approximately 3.7%, compared to the mortality rate of less than 1% from influenza [1]. As the number of those affected by novel COVID-19 increases, globally, so does the urge to find an appropriate pharmaceutical intervention. Antimicrobials and antivirals are at the center of the current exploration for the appropriate treatment. Many of the agents currently being tested through clinical trials are pre-existing medications that have been a part of the current market. These medications are being tested in hopes that they can be repurposed and with an adequate dose, inhibit either viral replication or inhibit host cell entry.

The global pandemic related to SARS-CoV-2 originated in Wuhan, China in December 2019 and was thought to have a zoonotic transmission with bats being the reservoir host. SARS-CoV-2 is an enveloped virus with a large positive-stranded RNA genome. On the surface of the virus is a spike protein; a type I membrane glycoprotein that constitutes peplomers and plays an integral role in the initiation of viral infectivity [2]. The spike protein is responsible for binding to the angiotensin-converting enzyme 2 (ACE2) receptor through which the virus gains entry into the type II pneumocyte present in the alveolar wall of the respiratory system. Upon binding to the ACE2 receptor, SARS-CoV-2 is endocytosed into the cytoplasm of the pneumocyte where the lysosomal enzymes of the host cell will break down the lipid bilayer of the virus, a process that is known as uncoating. The virus will utilize the host cell RNA dependent RNA polymerase to replicate its viral genome, increasing the viral load within the host cell [3]. Once the viral genome and the structural proteins have been replicated within the type II pneumocyte, the SAR-CoV-2 will bud off of the cell and in the process of budding off, destroying the pneumocyte. The destruction of the type II pneumocytes causes monocytes and macrophages to release cytokines such as interleukin-1 (IL-1), interleukin-6 (IL-6), and tissue necrosis factor-alpha (TNF-α). The increased release of cytokines causes systemic manifestations such as the presentation of fever, acute inflammation, smooth muscle dilation as the cytokines reach the systemic circulation. The cytokine storm leads to systemic inflammatory response syndrome (SIRS) in which systemic manifestations can lead to multi-system organ failure (MSOF) [2, 3]. Many pharmaceutical options target the various steps in the lifecycle of SARS-CoV-2, including viral entry. Many drugs show promise in the management of COVID-19, however, no pharmaceutical approach has been solidified. This manuscript aims to summarize the emerging pharmacological interventions for COVID-19, the mechanisms of action, and the adverse effects that are currently being researched.

## Review

There is an increased amount of pressure prevalent within the scientific and medical communities in attempting to find a proper medical strategy in managing and treating COVID-19. Several drugs are currently being researched that seem to be promising in the treatment of COVID-19, however, it should be noted with caution that these medical interventions are still being researched as no one approach has been solidified. Several antimicrobials and antivirals are currently being researched and investigated as they inhibit various steps within the lifecycle of SARS-CoV-2, as will be discussed. Supplements such as vitamin C and zinc are also under trial. While some of these pharmaceutical agents are administered as an independent dose, multiple drugs are co-administered. Although the mechanism of action amongst several agents may be similar, the adverse effects and the recommended dosing differ substantially. For each pharmaceutical agent reviewed, there will be an extensive focus on the mechanism of action and adverse effects along with any dosing recommendations that may have been explored.

Camostat mesylate

Camostat mesylate is a potent serine protease inhibitor that is approved for the treatment of pancreatic inflammation in Japan [4]. SARS-CoV-2 entry into the cell depends on the viral spikes protein to cellular receptors, angiotensin-converting enzyme 2 (ACE2), and S protein priming by host proteases (TMPRSS2) [5]. Camostat mesylate has recently been shown to block SARS-CoV-2 entry into the cell by inhibiting the cellular host TMPRSS2 [6]. With direct inhibition of entry into the cells, SARS-CoV-2 is unable to replicate as the virus requires the host's RNA-dependent RNA polymerase. Without access to adequate machinery, an infected individual may present as more of an asymptomatic carrier. Camostat mesylate is currently undergoing further clinical trials to fully understand the mechanism of action and if this protease inhibitor would prove to be a viable option in the treatment of COVID-19.

Arbidol

Arbidol (also known as Umifenovir) is a promising repurposed antiviral agent with a unique mechanism of action targeting the S protein/ACE2 interaction and inhibiting membrane fusion of the viral envelope to the host cell [7]. Arbidol is an antiviral drug that particularly focuses on inhibiting the fusion of the virion and the type II pneumocyte. Arbidol is a Russian-made potent broad-spectrum antiviral that has an established mechanism of action against influenza A and V viruses [8]. Arbidol is better known in China and Russia and to a lesser extent in western countries. The predominant mode of action of arbidol is based on its intercalation into membrane lipids leading to the inhibition of membrane fusion between virus particles and plasma membranes, and between virus particles and the membranes of endosomes [9]. The efficacy and safety of arbidol monotherapy were analyzed and on day 14 after admission, no viral load was detected [10]. Arbidol has been noted as a well-tolerated molecule with a high therapeutic index, when administered on periods ranging from a few days to a month, however, no studies have addressed the long-term administration of arbidol, such as in the context of chronic infections [11]. The maximum blood concentration of arbidol occurs 1.2 to 1.5 hours after the administration of a dose and the half-life of the drug in the body is 17 to 21 hours [12]. Arbidol is metabolized in the body and is excreted mostly through bile and an insignificant amount through kidneys [11,12]. Arbidol is typically well-tolerated and has low toxicity, however, the main adverse effects include nausea, diarrhea, dizziness, and elevated serum transaminase [12].

Chloroquine/Hydroxychloroquine

Currently, chloroquine/hydroxychloroquine is one of the most promising compounds that has gained international attention for its potential activity against COVID-19 [6]. Chloroquine is active against malaria as well as amongst many autoimmune diseases, such as systemic lupus erythematosus or rheumatoid arthritis. Several clinical trials in China have shown chloroquine phosphate to be effective against COVID-19 at a dose of 500 mg/day [13]. Hydroxychloroquine is reported to be as active as chloroquine, however less toxic [6]. Due to the increased toxicity concerns that limit the use of chloroquine, a compound that differs from chloroquine by a single hydroxyl group, hydroxychloroquine, is used [14]. Chloroquine was shown to increase endosomal pH, which presents virus/cell fusion and it also interferes with the glycosylation of cellular receptors of SARS-CoV [15]. Increasing the pH of the lysosome prevents protease activity such that the fusion process is disrupted [16]. Chloroquine has been shown to exert an antiviral effect during pre- and post-infection conditions by interfering with the glycosylation of the ACE2 receptor thus, reducing the binding efficiency between ACE2 on host cells and the SARS-CoV-2 spike protein [13]. While the drug was shown to inhibit the virus in vitro, the required dose in humans is thought to be quite high and lead to severe toxicity [6]. Both chloroquine and hydroxychloroquine are also said to have immunomodulatory effects through attenuation of cytokine production and inhibition of autophagy and lysosomal activity in host cells [7]. The cytokine production in COVID-19 occurs when the replicated virion is budding off of the type II pneumocyte and is responsible for many systemic processes such as fever, and various other processes that ultimately can lead to the fatal complication of acute respiratory distress syndrome. Inhibiting the cytokine production would be beneficial to prevent the progression of the viral disease, however, the adverse reactions become a significant obstacle that needs to be overcome. While the use of chloroquine and hydroxychloroquine is considered safe in pregnancy, the adverse effects are plenty as they range from QT prolongation, hypoglycemia, neuropsychiatric effects and retinopathy [7,17].

Lopinavir and ritonavir

Lopinavir is a human immunodeficiency virus (HIV)-1 protease inhibitor administered in fixed-dose combination with ritonavir, a potent CYP3A4 inhibitor that ‘boosts’ lopinavir concentrations [14]. The use of this combination was shown to have potent antiviral activity against the severe acute respiratory syndrome (SARS) virus [6]. Lopinavir is a highly potent and selective inhibitor of the HIV type 1 (HIV-1) protease, an essential enzyme for the maturation of the virus [18]. Inhibiting the enzyme arrests the maturation of the virus and thus, blocking its infectivity. Without the protease function, only immature, non-infectious viral particles are formed, thus attenuating the viral load of SARS-CoV-2 amongst infected patients. Enzyme 3-chymotrypsin-like protease (3CLpro) plays a crucial role in processing the viral RNA and with lopinavir administration, it has been postulated that lopinavir may inhibit the action of 3CLpro, disrupting the process of viral replication and release from host cells [19]. Although lopinavir does not affect the already infected cells, the main antiviral action of the protease inhibitor is to prevent subsequent infection of cells. Ritonavir is administered in a lower dose than lopinavir and in doing so, the co-administration inhibits metabolic inactivation of lopinavir and acts as its pharmacokinetic enhancer [7,18]. Lopinavir is a cytochrome P450 inhibitor and thus, drug interactions with lopinavir/ritonavir are fairly common. The treatment of lopinavir-ritonavir was shown to be effective as post-exposure prophylaxis against other viral diseases including middle east respiratory syndrome (MERS) [6]. A study compared 111 patients with the severe acute respiratory syndrome (SARS) treated with ribavirin monotherapy and 41 patients with SARS treated with lopinavir/ritonavir and ribavirin; patients treated with the combined therapy had a lower risk of acute respiratory distress syndrome (ARDS) and death [20,21]. In 2003, Chu et al. evaluated that lopinavir at 4 µg/mL and ribavirin at 50 µg/mL inhibited SARS-CoV-1 after 48 hours of incubation and that the agents were synergistic when used together [20]. The most frequent side effects observed are gastrointestinal, such as diarrhea, nausea, and vomiting [22]. Lopinavir is primarily metabolized by the liver and thus, drug concentrations may increase in individuals with hepatic impairment or slow acetylators. The timing of administration during the early peak viral replication phase (initial 7-10 days) appears to be important because delayed therapy initiation with lopinavir/ritonavir did not affect clinical outcomes [7,23].

Ivermectin

Ivermectin has been identified very largely in the search for appropriate pharmaceutical intervention in COVID-19. Ivermectin is an FDA-approved broad-spectrum antiparasitic agent that in recent years, has shown to have antiviral activity against a broad range of viruses in vitro [24]. Ivermectin possesses the ability to disassociate the preformed IMP α/β1 heterodimer which is responsible for the nuclear transport of viral protein cargos [25]. The inhibition of IMP α/β1 disrupts the immune evasion mechanism of the virus [24]. Studies on SARS-CoV proteins have revealed a potential role for IMP α/β1 during infection in the signal-dependent nucleocytoplasmic shutting of the SARS-CoV nucleocapsid protein that may impact host cell division [26]. The exact mechanism behind ivermectin is still under investigation but studies have found that a single treatment of ivermectin in a COVID-19 patient can cause the effect of a ~5000-fold reduction in viral RNA at 48 hours [24,26]. Due to an immensely significant decrease in viral load with just one dose, ivermectin holds a significant amount of promise and thus, warrants further investigation in humans.

Ribavirin

Ribavirin, a guanine analog, inhibits viral RNA-dependent RNA polymerase [7]. Ribavirin was first approved in the 1980s and has been used clinically for the respiratory syncytial virus, viral hemorrhage fever, and in combination with interferon for hepatitis C [14]. By inhibiting RNA-dependent RNA polymerase, ribavirin inhibits the initiation and elongation of RNA fragments, resulting in the inhibition of viral protein synthesis [27]. To further promote the destabilization of viral RNA, ribavirin inhibits natural guanosine generation by directly inhibiting inosine monophosphate dehydrogenase in a pathway that is vital for the production of guanine precursor to guanosine [28]. Guanosine is necessary for the capping of RNA to prevent degradation. Without proper guanosine available, RNA degradation will more readily occur and thus, decreasing the viral load. In the case of SARS-CoV-2, the virion genome will be destabilized due to the missing guanosine cap. It should be noted that these various mechanisms of action of ribavirin are dose-dependent, however, currently, there is no knowledge regarding which dose will cause which effect [21,28]. Ribavirin should be administered via intravenous infusion at a dose of 500 mg for adults, 2 to 3 times/day in combination with interferon-α or lopinavir/ritonavir for no more than 10 days [21]. The in vitro activity against SARS-CoV was limited and required high concentrations to inhibit viral replication, necessitating high dose, and combination therapy [7]. The high doses of ribavirin used in the SARS trials resulted in hemolytic anemia in more than 60% of patients, resulting in a severe dose-dependent hematologic toxicity [7,27]. The adverse effect of anemia is seen particularly at the dosages for which it has been tested for MERS (~800-3600 mg/day) [27]. With the increased risk of anemia at the doses necessary, ribavirin should not be implicated as the first drug of choice in COVID-19 patients. Ribavirin is also a known teratogen and contraindicated in pregnancy [7]. This contraindication should be tested further to understand the exact effects of teratogenicity.

Remdesivir

Remdesivir, a broad-spectrum antiviral, is another potential drug that is under investigation for the treatment of COVID-19. Animal experiments indicate that remdesivir can effectively reduce the viral load in lung tissue of mice infected with MERS-CoV, improve lung function, and alleviate pathological damage to lung tissue [29]. Remdesivir is an investigational monophosphoramidate prodrug of an adenosine analog that, in its active triphosphate nucleoside form, binds to RNA-dependent RNA polymerase and acts as an RNA-chain terminator against the Ebola virus [14]. The first clinical use of remdesivir was for the treatment of Ebola, however, successful case reports describing the use of remdesivir for COVID-19 have been reported [7,30]. Remdesivir is also highly selective for viral polymerases and is therefore expected to have a low propensity to cause human toxicity [14]. Remdesivir is metabolized into its active form, GS-441524, that obscures viral RNA polymerase and evades proofreading by viral exonuclease, causing a decrease in viral RNA production [31]. In vitro studies with mice, hepatitis virus showed that remdesivir inhibits coronavirus replication through interference with the viral polymerase, despite the presence of a viral proofreading exoribonuclease [32]. The first case of COVID-19 in the United States was noted to be in Washington. This patient was compassionately treated with intravenous remdesivir for the progression of pneumonia on day 7 of hospitalization [30]. The patient’s condition had notably improved and no obvious adverse effects were observed [33]. Despite the nasopharyngeal and oropharyngeal swabs remaining positive four days following the administration of remdesivir, authors had noted a trend in the decline of viral load [30,33]. The 10-day regime of remdesivir treatment consists of an initial dose of 200 mg on day 1, followed by subsequent doses of 100 mg for nine consecutive days via intravenous infusion [21,33]. A trial sponsored by the National Institute of Allergy and Infectious Diseases (NIAID) presented with promising preliminary results. The trial consisted of 1063 patients and had initially begun on February 21, 2020. Preliminary results indicate that patients who received remdesivir had a 31% faster time to recovery than those who received a placebo and furthermore, the median time to recovery was 11 days for patients treated with remdesivir compared with 15 days for those who received the placebo [34]. With such promising results, remdesivir is currently being referred to as the standard of care for COVID-19.

Favipiravir

Favipiravir is a prodrug of a purine nucleotide, favipiravir ribofuranosyl-5’-triphosphate that inhibits the RNA polymerase, halting viral replication [7]. In addition to its anti-influenza virus activity, favipiravir is capable of blocking the replication of flavi-, alpha-, filo-, bunya-, arena-, novo-, and other RNA viruses [21]. A loading dose is recommended (2400 to 3000 mg every 12 hours x 2 doses) followed by a maintenance dose (1200 mg to 1800 mg every 12 hours) [7]. Favipiravir has mild adverse effect profile and is overall well-tolerated, although the adverse event profile for higher dose regimens is limited [7,35]. The exact antiviral effect of favipiravir in patients with COVID-19 needs demanding data to support as several clinical trials are evaluating this agent in different combination regimens [27].

Tocilizumab and sarilumab

Tocilizumab, as immunotherapy, is a humanized anti-interleukin 6 monoclonal antibody for the treatment of rheumatoid arthritis [7,27]. It inhibits both membrane-bound and soluble interleukin-6 (IL-6) receptors [14]. COVID-19 patients present with hyperinflammatory states and cytokine storms that include elevated levels of IL-6. IL-6 is one of the several cytokines that are released by both monocytes and macrophages in response to destruction to the type II pneumocytes. Type II pneumocytes are destroyed as the replicated virion genome buds off of the pneumocyte. IL-6 is a cytokine that is a key player in causing fever and acute inflammation. Currently tocilizumab is being tested in patients with the potential risk of cytokine storm-driven hyper inflammation (also known as cytokine release syndrome) that can be triggered by infections, including COVID-19 [6]. Given the experience, tocilizumab has been used in a small series of severe COVID-19 cases with early reports of success [7]. A report of 21 patients with COVID-19 showed receipt of tocilizumab, 400 mg, was associated with clinical improvement in 91% of patients as measured by improved respiratory function, rapid defervescence, and successful discharge, with most patients only receiving one dose [36]. A preprint (nonpeer reviewed) case series of 21 patients treated with tocilizumab between February 5 and 14, 2020 in China reported marked success, including rapid resolution of fever and C-reactive protein, decreased oxygen requirements, and resolution of lung opacities on computerized tomography imaging [7]. Tocilizumab presents with promising effects as depicted in various trials surrounding COVID-19. The recommended dose is 4-8 mg/kg or 400 mg standard dose IV once, with the option to repeat a dose in 12 hours [7,36]. The optimal timing of tocilizumab administration during the disease course is not yet defined, nor is there a known IL-6 threshold for progression to severe disease [7]. There were no reported adverse events in the 60 tocilizumab-treated patients submitted to the FDA [37]. Sarilumab, another IL-6 receptor antagonist that has been approved for use in rheumatoid arthritis, is being studied for hospitalized patients with severe COVID-19 [7].

Oseltamivir and baloxavir

Given their antiviral activity against influenza, considerable attention has been paid to oseltamivir, and a lesser degree baloxavir, as potential treatment options for COVID-19 [14]. Oseltamivir is a neuraminidase inhibitor that is approved for the treatment of influenza, however, it has no documented in vitro activity against SARS-CoV-2 [7]. The correlation of oseltamivir and baloxavir with COVID-19 is purely coincidental. The COVID-19 outbreak in China initially occurred during peak influenza leading to a large proportion of patients received empirical oseltamivir therapy until the discovery of SARS-CoV-2 as the cause of COVID-19 [38]. Neither oseltamivir nor baloxavir plays any role in the management of COVID-19 in cases where influenza has been excluded or is not comorbid.

## Conclusions

As the outbreak of COVID-19 continues to create fear within the medical community, investigating all possible pharmaceutical options is necessary. Several different pharmacological options are constantly emerging and looking into each of the interventions is crucial in attempts to contain the current pandemic. Currently, there are no fully verified antiviral drugs or vaccines that are specific to fighting SARS-CoV-2. Many pharmaceutical approaches being currently explored are those that have been used for various different conditions. The repurposing of these drugs provides hope that a cocktail of drugs currently on the market may appropriately target various steps in the lifecycle of SARS-CoV-2, allowing for the ultimate degradation of the virus. Decreasing the viral load by destabilizing the RNA-dependent RNA polymerase will hinder the replication of the virion, and ultimately, decreasing the infectivity of SARS-CoV-2. Other approaches focus on impairing the fusion of SARS-CoV-2 with the type II pneumocytes ACE2 receptor. Inhibiting fusion prevents any type of destruction of the type II pneumocytes, eliminating any type of infectivity of the virus. It should be noted that almost all the pharmaceutical approaches are currently being further researched through clinical trials. These clinical trials will ultimately provide insight into the efficacy and safety of these candidate drugs, thus providing the medical field with appropriate alternatives to treating those infected by this pandemic.
